# The function of omega-3 polyunsaturated fatty acids in response to cadmium exposure

**DOI:** 10.3389/fimmu.2022.1023999

**Published:** 2022-09-29

**Authors:** Zhi Chen, Qinyue Lu, Jiacheng Wang, Xiang Cao, Kun Wang, Yuhao Wang, Yanni Wu, Zhangping Yang

**Affiliations:** ^1^ College of Animal Science and Technology, Yangzhou University, Yangzhou, China; ^2^ Joint International Research Laboratory of Agriculture & Agri-Product Safety, Ministry of Education, Yangzhou University, Yangzhou, China; ^3^ College of Medical, Yangzhou University, Yangzhou, China

**Keywords:** cadmium, omega-3 PUFAs, immune system, oxidative stress, health

## Abstract

Throughout history, pollution has become a part of our daily life with the improvement of life quality and the advancement of industry and heavy industry. In recent years, the adverse effects of heavy metals, such as cadmium (Cd), on human health have been widely discussed, particularly on the immune system. Here, this review summarizes the available evidence on how Cd exposure may affect health. By analyzing the general manifestations of inflammation caused by Cd exposure, we find that the role of omega-3 (n-3) polyunsaturated fatty acids (PUFAs) *in vivo* can counteract Cd-induced harm. Additionally, we elucidate the effects of n-3 PUFAs on the immune system, and analyze their prophylactic and therapeutic effects on Cd exposure. Overall, this review highlights the role of n-3 PUFAs in the pathological changes induced by Cd exposure. Although n-3 PUFAs remain to be verified whether they can be used as therapeutic agents, as rehabilitation therapy, supplementation with n-3 PUFAs is reliable and effective.

## Introduction

Heavy metals exist everywhere. It is dangerous to consume excessive amounts of any metal, including those normally found in the environment, such as zinc, copper, and iron, as well as discolored metals like lead, arsenic, mercury, and cadmium (1). Among them, cadmium (Cd), upon entering the body, could cause huge damage to a series of important organs, as well as the nervous system, reproductive system, immune system, and other systems. Additionally, it has strong toxic effects on cells. Short-term exposure to Cd can cause apoptosis and necrosis in cells, and the long-term exposure can induce cancerous cells and lead to tumors. So far, only chelation therapy has proven to be effective for Cd poisoning (2) (**Table 1**).

**Table 1:** Common cadmium exposure treatment options.

Treatment Method	Apply	Defect
Chelating agents		
Ethylenediaminetetraacetic acid (EDTA)	Significantly increases the elimination of cadmium in urine	May increase Cd levels in the kidneys and may increase the risk of renal insufficiency
Dimercaprol	Used as an antidote for heavy metal poisoning	Must be administered within the first 4 hours of intoxication and increases risk of nephrotoxicity
Dithiocarbamates	Increased urobiliary excretion of cadmium	There is currently no complete treatment plan
Meso 2, 3-dimercaptosuccinic acid (Succimer, DMSA)	Water-soluble analogs of dithiocarbamates	Non-intracellular chelating agent, the effect is not obvious
**Combination therapy**	The combination of DMSA and MiADMSA can have a good therapeutic effect	Increased risk of nephrotoxicity
**Plasma exchange**	Helps with heavy metal toxicity	Not recommended for use in non-emergency situations
**Hemodialysis**	Relieve kidney burden and protect kidney function	Almost ineffective for cadmium elimination

In view of the fact that oxidative stress is one of the necessary mechanisms of Cd-induced damage, the administration of some antioxidants is expected to be a crucial therapeutic approach. In rehabilitation therapy, omega-3 (n-3) polyunsaturated fatty acids (PUFAs), which have been shown to respond to oxidative stress in the body, are widely used as antioxidants and anti-inflammatory agents. Besides, some existing studies have pointed out n-3 PUFAs could be a possible treatment for Cd exposure in the body (3–5), thus it is necessary to explain and analyze their mechanisms for further advancement of relevant research.

## The protective function of n-3 PUFAs

**Figure 1:**
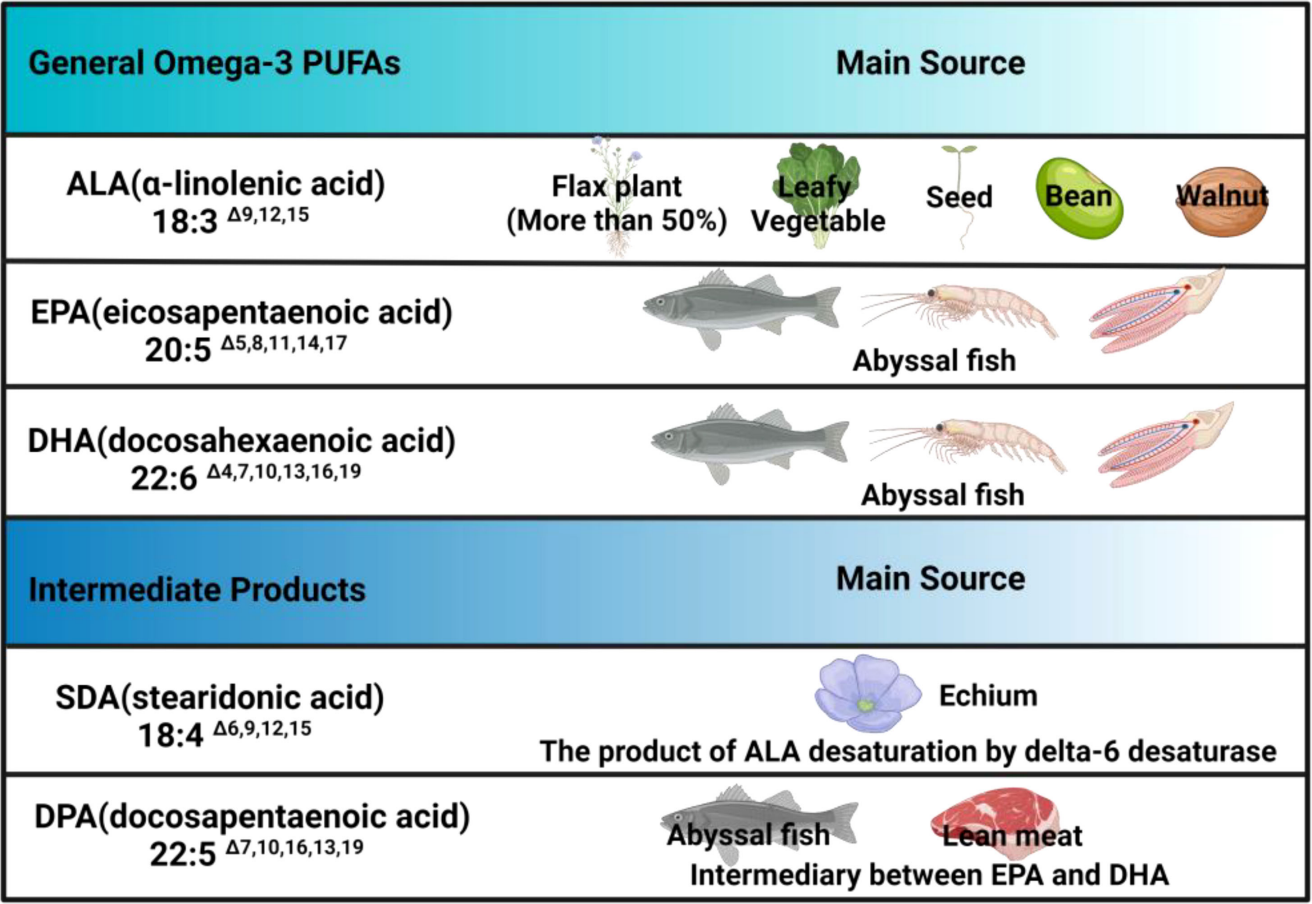
Omega-3 PUFAs and their main sources. N-3 PUFAs mainly consist ALA, EPA, DHA and intermediate derivatives, among which only SDA and DPA are listed in the table, but other derivatives, such as eicosatetraenoic acid (ETA), are not included, which means there is a lack of clarity regarding these derivatives currently.

More than 15,000 studies have shown that n-3 PUFAs are anti-inflammatory, regulating the immune system and showing a wide range of beneficial benefits in mammals. Generally, fatty acids (FAs) can be divided into short-chain, medium-chain, long-chain, and very-long-chain FAs. According to the degree of unsaturation on the carbon chain, they are also divided into saturated FAs, monounsaturated FAs (containing one double bond), and polyunsaturated FAs (containing multiple double bonds) (6, 7). As nutrition has developed, studies have found that FAs with double bonds in different positions have different nutritional value. Thus, they are classified into n-3 PUFAs and omega-6 (n-6) PUFAs according to the position of the last double bond relative to the methyl terminal of the molecule. Among them, n-3 PUFAs mainly include α-linolenic acid (ALA; 18:3 n-3), eicosapentaenoic acid (EPA; 20:5 n-3), and docosahexaenoic acid (DHA; 22:6 n-3). In addition, stearidonic acid (SDA, 18:4 n-3) and docosapentaenoic acid (DPA, 22:5 n-3) are intermediates of n-3 PUFAs that have attracted extensive attention. Among them, DPA is an intermediate product between EPA and DHA, and SDA, derived from ALA, exists in the biosynthetic pathway of DPA and DHA (**Figure 1**).

### In vivo

EPA and DHA are two forms of n-3 PUFAs that are widely distributed in mammals as essential FAs (8). In order to prolong and transform, it is necessary for n-3 PUFAs to enter the gastrointestinal tract and combine with chylomicrons before being transferred into the liver as glycerol (9). The liver processes ALA and EPA into docosapentaenoic acids (DPA, an n-3 fatty acid) and DHA by desaturases and fatty acid chain elongases, but they cannot be processed unlimitedly. Studies have shown that platelet membranes are involved in regulating the concentrations of DHA (10, 11). When DHA is saturated, they even promote the reverse conversion of excess DHA to EPA and DPA which are then bound to triglyceride in the form of very low-density lipoprotein cholesterol and release it into the blood. Subsequent studies have shown that the DHA content in the platelet membrane may be responsible for mechanism of n-3 PUFAs (12). In mice, highly purified DHA could significantly increase DHA, DPA, and EPA contents in the platelet membrane, while highly purified EPA could only increase EPA and DPA contents (13). This again proves that DHA supplementation is effective in rehabilitation therapy or treatment. In addition, n-6 PUFAs also affect the metabolism of n-3 PUFAs (14–16), since liver is the main site of desaturations and elongations of n-3 PUFAs and n-6 PUFAs. Their metabolisms are based on the same enzymes, which makes them competitive. There is evidence that higher concentration of n-6 PUFAs diet could result in lower conversion rate of ALA, and conversely, high concentrations of ALA could also reduce conversion of n-6 PUFAs (17–19). In light of this, numerous studies have revealed that the ratio of n-3/n-6 is closely related to body health, which again highlights the importance of the ratio of healthy dietary intake.

### In vitro

In the immune system, n-3 PUFAs are found in almost all known immune cells, among which the macrophages are the most commonly mentioned. To date, the effects of n-3 PUFAs on the production and secretion of cytokines and chemokines by macrophages have extensively been investigated, as well as the regulatory mechanisms behind them. As part of the innate immune system, macrophages have the function of locating pathogens. Studies have found that n-3 PUFAs can participate in regulating the production and secretion of cytokines and chemokines in macrophages, thus having the phagocytic ability of macrophages (20). In addition, some studies have also pointed out that n-3 PUFAs could change the activation state of macrophages (21–23). In the course of research, n-3 PUFAs are found to play an important role in neutrophils, which is related to the n-3/n-6 competition. When n-3 is heavily involved in the phospholipid composition of neutrophil membrane, n-6 PUFAs will be metabolized to other fatty acid derivatives with anti-inflammatory properties, such as prostaglandins and protectins (24–26). In addition to this, neutrophils rich in n-3 PUFAs are able to migrate better, which has also been validated *in vivo* (27–29). Interestingly, as metabolites derived from n-3 PUFAs appear to inhibit the migration ability of neutrophils, we speculate that this is the result of their metabolism. It is worth mentioning that when oxidative stress occurs in the body, there will be inflammatory infiltration of neutrophils, increase of proteases secretion, and the production of a large number of oxidative intermediates.

Moreover, n-3 PUFAs also seem to be present in oxidative stress. Studies have shown that supplementation of n-3 PUFAs could increase production of reactive oxygen species (ROS) in neutrophils (30–32). Although this performance appears to be age-related, we are more interested to know whether this phenomenon simply supplements the missing ROS or unrestricted production, which will have a substantial impact on our subsequent utilization of n-3 PUFAs. For the main lymphocytes in the immune system, many studies have demonstrated the role of n-3 PUFAs in T cells (**Table 2**) and B cells (**Table 3**), but many conclusions are not unified because they have different subgroups/populations. Thus, in this article, we only list the references without further analysis. Also, some other cells related to the immune system are regulated by n-3 PUFAs, such as eosinophils and basophils (56–59). However, they will not be investigated here because current research cannot form a complete system, instead we will discuss the experimental methods on n-3 PUFAs. In the future, more research will look at supplementation of EPA or DHA to observe changes in the body, but at this point it is unknown whether single or multiple PUFAs play a role. It may be necessary, therefore, to improve the current commonly used experimental methods to be supplemented alone or inhibited alone.

**Table 2:** Effects of n-3 PUFAs supplementation on T cells.

Type	Role	Reference
**General Effect**	Suppressive effect	(33, 34)
**CD4**	Prevent differentiation	(35, 36)
	Inhibit secretion of IFN-γ	(37)
	Inhibit secretion of IL-2	(38)
	Inhibit secretion of IL-17	(39)
	Reduce cell proliferation	(40)
**CD8**	Suppressive effect	(41, 42)
	Inhibit secretion of TNF-α	(43)
	Inhibit secretion of IFN-γ	(44)
	Inhibit secretion of IL-2	(45)
	Reduce cell proliferation	(44)

**Table 3:** Effects of n-3 PUFAs supplementation on B cells.

Type	Role	Reference	Remark
**The proportion of B cells**	Decreased numbers of naive B cells	(46)	
	Decreased numbers of mature B cells	(47)	
**The function of B cells**	Inhibit secretion of IL-6	(48)	
	Inhibit secretion of IL-10	(49)	
	Inhibit secretion of TNF-α	(50)	
	Inhibit secretion of IFN-γ	(51)	
	Up-regulates the expression of activation markers	(52)	contradictory
	Up-regulate the expression of IgM	(53–55)	contradictory

### Clinical aspects

**Figure 2:**
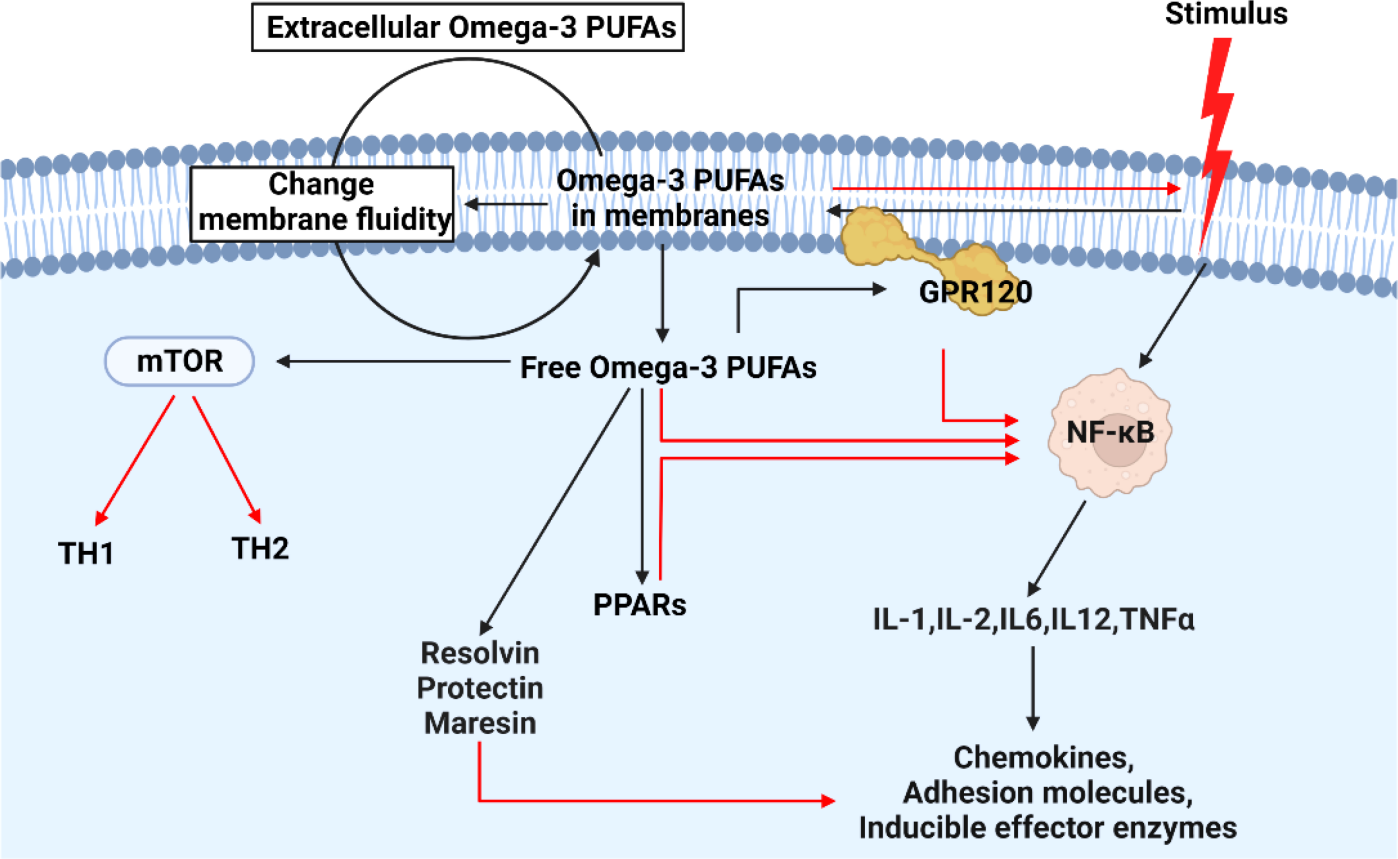
The mechanism of action of n-3 in cells when subjected to external inflammatory stimuli. When cells encounter external inflammatory stimuli, n-3 PUFAs will be activated. On the one hand, more extracellular n-3 PUFAs enter the cell by changing the fluidity of the cell membrane. On the other hand, free intracellular n-3 PUFAs inhibit the expression of intracellular inflammatory factors by combing with other factors. Among them, GPR120: free fatty acid receptor 4 (FFA4). In the figure, the red line indicates the inhibitory effect.

In what ways did n-3 PUFAs affect the harmful? There is evidence that the n-3 PUFAs can inhibit the inflammation by activating various genes and pathways including IL1, IL-2, IL-6, IL-12, TNF- α, peroxisome proliferator-activated receptors (PPARs), and so forth (60–64), through absorbing supplemented n-3 PUFAs. Also, some studies have delved into the pathway and found that n-3 PUFAs regulate these factors through a number of master transcription factors, including sterol regulatory element-binding protein (SREBP), PPAR, nuclear factor-kappa B (NF-κB), and carbohydrate response element-binding protein (ChREBP) (65–68) (**Figure 2**). In addition, intermediates formed by oxidative synthesis of n-3 PUFAs are also important, and dietary n-3 PUFAs are stored in the form of triacylglycerols and phospholipids. During the resolution of acute inflammation, the organism promotes the conversion of n-3 DHA into d-series resolvins and protectins/neuroprotectins (69). In macrophages, DHA is also converted to maresins (macrophage mediators in resolving inflammation) *via* 14-lipoxygenation (70). Moreover, these mediators are capable of limiting neutrophil recruitment and potently stimulating macrophage phagocytosis of apoptotic cells in a stereospecific manner. Aside from that, neuronal transient receptor potential vanilloid 1 (TRPV1) currents are also inhibited by them, which regulates inflammation and chemotherapy-induced pain.

In conclusion, normally n-3 PUFAs have a regulatory effect on immune cells of the innate and adaptive branches. Here, the ability of n-3 PUFAs in controlling the inflammatory response and switching inflammation into a regressive state is emphasized, which is the basis to combat inflammation caused by Cd exposure. Additionally, many mechanisms have not been fully elucidated, such as whether n-3 PUFAs play a role in relation to cellular localization and metabolic inactivation pathways. With the advent of new technologies such as molecular imaging and lipidomics, it may not be too late to reveal how these mechanisms work.

## The possible harm of Cd exposure to the body

**Figure 3:**
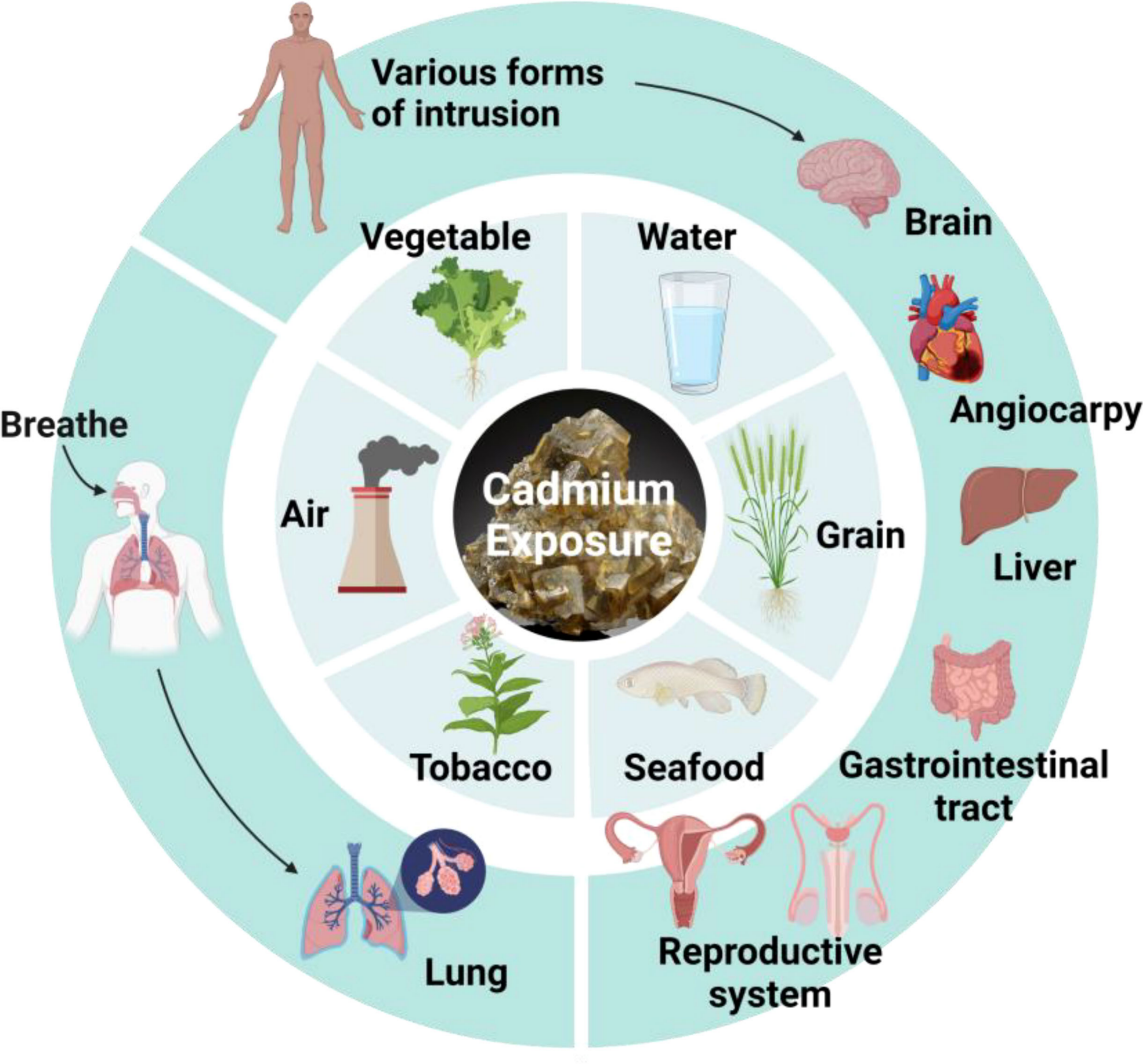
Hazards from Cd exposure. Cd can enter the body through air, tobacco, seafood, grain, water, vegetable, and damage different organs.

Cd, a toxic metal, poses a health risk to both humans and animals (**Figure 3**). In fact, Cd exposure occurs naturally in the environment since Cd is a pollutant from agricultural and industrial sources. In addition to inhalation by smoking and pulmonary ingestion (71), studies have indicated that Cd can also be ingested by food (72). Although only 5% of Cd in food is absorbed into the human body through the gastrointestinal tract, it is much lower than the absorption capacity of the lungs. Ingestion through food, however, is the most significant route of exposure for general non-occupational populations. In addition, Cd can be ingested through skin contact (73). Although this kind of penetration is not ideal, there is still some risk.

### Liver

After Cd enters the human body, it can cause damage to a series of important organs such as liver, kidney, bone, brain and lung, as well as nervous system, reproductive system, immune system, and other systems, among which liver and kidney are the most important target organs. It is reported that the toxicity of Cd is obviously time-dependent and concentration-dependent (74–77). The Cd-induced liver injury is mainly due to the competitive replacement of Cd with the metal prosthetic groups in the liver antioxidant enzymes, which inactivates the antioxidant enzymes and reduces the scavenging ability of free radicals, thereby causing cellular lipid peroxidation and oxidative stress (78, 79). In addition, the entry of Cd can also cause cells to secrete pro-inflammatory factors and chemokines, resulting in apoptosis and pathological changes, and eventually the formation of tumors. A study found that acute Cd poisoning could bring about an increase in the level of a series of proteases related to liver in the blood, which in turn results in the incidence of non-alcoholic hepatitis and fatty liver (80).

### Kidney

Low concentrations or chronic Cd poisoning mainly cause kidney damage. In clinical manifestations, renal tubular reabsorption dysfunction occurs in the early stage of Cd poisoning, and low molecular weight proteins appear in the urine (81–83). With the aggravation of Cd damage to the kidney, macromolecular proteinuria would occur in the body. At the same time, a large quantity of proximal convoluted tubule cells undergo apoptosis, and the activities of Na/K-ATPase and other proteins decrease significantly. In addition, the glomerulus and distal convoluted tubules are found to be affected in different degrees. It is worth noting that the damage caused by Cd to the kidney tissue is extremely difficult to repair on account of urinary physiology (84, 85). The Cd would travel to the kidneys with the blood and filter through the glomeruli, and then almost all of Cd would be reabsorbed by the proximal convoluted tubule with only trace amounts of them could be excreted. And Cd in excess of the treatment range will severely damage the function of the glomerulus, bringing about a series of health problems. Studies have pointed out that the kidneys will undergo fibrosis and even necrosis if glomeruli fail to function (86, 87).

### Bone

In addition, patients in incident of Cd-contaminated water source in Japan in the 1960s suffered from some pathological injuries, including bone pain, susceptibility to fractures, developmental deformities of long bones, osteoporosis, osteomalacia, and so on (88). It is reported that these damages are related to kidney disease with a large number of calcium ions being excreted from urine, thus affecting the growth and development of bones (89, 90). Aside from that, Cd can directly damage bones by inhibiting the differentiation of osteoblasts and promoting the formation and function of osteoclasts, leading to disorder in the normal bone formation and pathological damage of bones (91).

### Other organs

In addition to liver, kidney and bones, exposure to Cd can also cause serious damage to other organs. As a result of Cd’s serious neurotoxicity, it can cause a series of neuropathy throughout the body. A study found that the lesions are mainly caused by embryonic transmission and respiratory pathways (92–95). Similarly, in addition to contributing to toxic effects on the peripheral nervous system, the absorbed Cd also damages olfactory neurons, central nervous cells, and oligodendrocytes through competitive replacement, as well as generates a large number of free radicals after entering the brain, which can induce many diseases including headache, dizziness, olfactory dysfunction, Parkinson-like symptoms, vasomotor control impairment, and learning impairment. Notably, this mode of transmission could bring more damage to babies whose immune systems are not yet fully developed. Numerous studies have shown that Cd damages the central nervous system in infants at lower doses than in adults, which is also related to a weaker blood-brain barrier in infants (96, 97).

In addition, people involved in the Cd industry frequently suffer from respiratory and lung diseases, such as chronic rhinitis, pharyngitis, pneumonia, pulmonary fibrosis, and emphysema (98–100). As another target of Cd, the gastrointestinal tract is also affected, and Cd exposure could cause gastrointestinal cell apoptosis, intestinal tissue villi damage, and changes in the structure of the intestinal microbiome (101–103). In the cardiovascular aspect, contrary to previous studies that did not attribute to Cd on this aspect, recent studies have shown that Cd poisoning can cause vascular inflammation, promote vascular arteriosclerosis, damage the structural integrity of blood vessels, and diminish myocardial contractility and cardiac conduction excitation (104–107). A series of conditions can also occur, such as the decline in libido and coronary blood flow. Although mechanism of Cd action has not been fully elucidated, there is no doubt that it causes damage to cardiovascular system.

Also, certain pathological changes, including cancer and other chronic diseases, can be associated with Cd exposure (108). Several studies examining Cd exposure suggest that Cd is responsible for some malignancies, such as pancreatic cancer (109), and both human and mouse models have indicated a link between Cd and pancreatic cancer (110, 111). Another cancer thought to be induced by Cd is kidney cancer (112), which is associated with kidney damage as previously described, as Cd can cause a wide range of pathologies in renal tissue from renal insufficiency to renal cancer. In addition, despite several studies suggesting that heavy metals are involved in the development of breast cancer (113), more research is needed to prove whether Cd is involved. According to studies, breast cancer tissues accumulate large amount of Cd after Cd exposure, and DNA methylation levels correlate positively with cadmium exposure (114), whereas in a study on humans, Cd levels in diet and urine do not appear to be correlated with breast cancer incidence and mortality (115). Aside from that, research has determined that Cd contributes to diabetes occurrence (116, 117). By acting on islet β cells, up-regulation of inflammatory factors (such as TNF-α,IL-1,IL-6) significantly increases the incidence of diabetes by inducing intracellular lipid accumulation and affecting its insulin secretion function.

**Figure 4:**
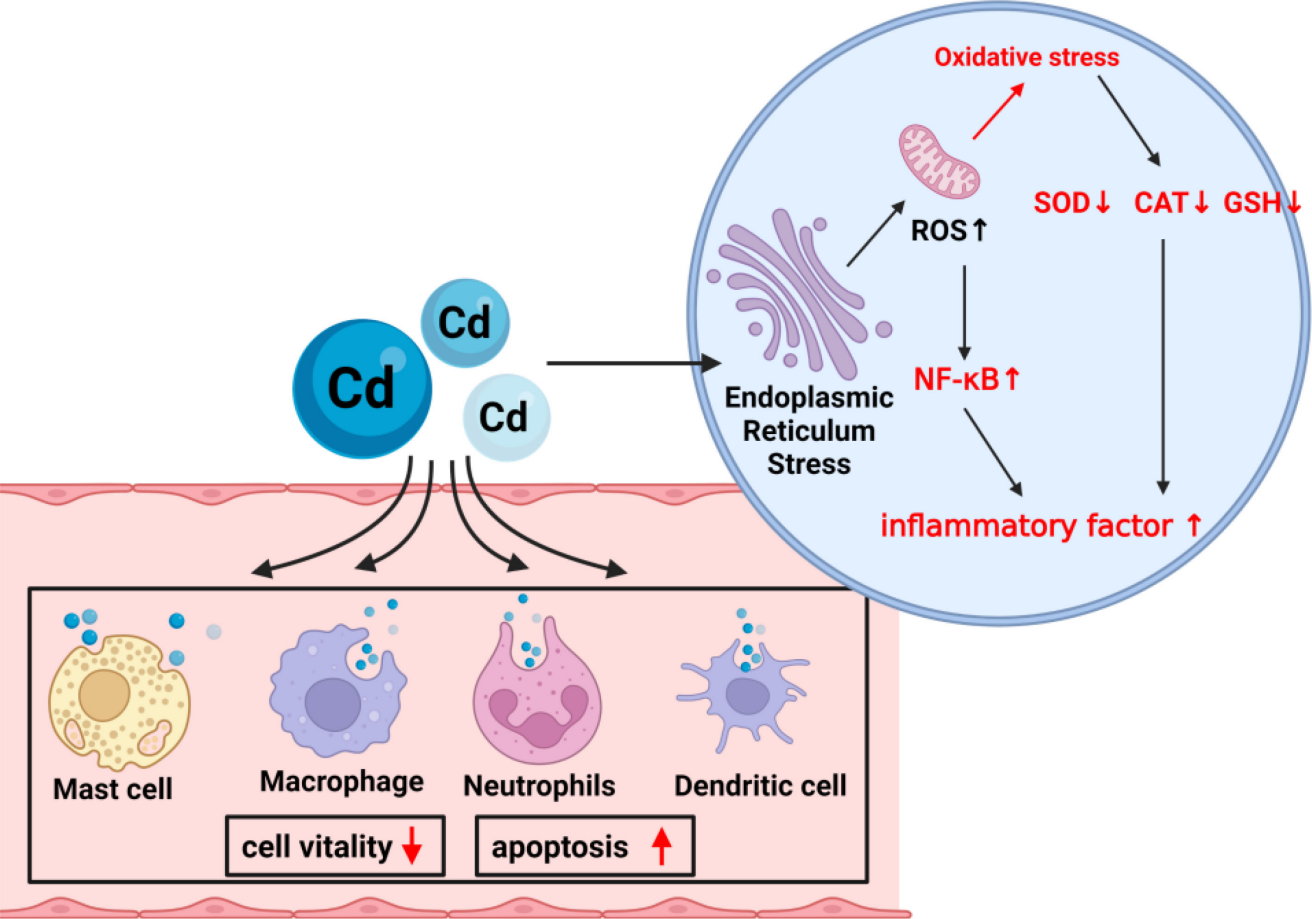
The mechanism of Cd exposure on cells. Cd exposure damages most of the immune cells in the immune system, including decreased vitality and increased apoptosis rate. Oxidative stress in cells occurs mainly through damage to the endoplasmic reticulum and mitochondria.

As an immunotoxic inhibitor, Cd interacts with almost all immune cells, impairing the immune system in a time- and dose-dependent manner (**Figure 4**). In summary, its toxic effects are achieved by competitive replacement of proteases (essentially replacing essential metals in proteases), thereby inducing apoptosis or oxidative stress. So far, most studies on Cd exposure have focused on exploring the effects of Cd ions on the body, but few have examined its toxic mechanism and corresponding potential detoxification mechanism. An urgent need is to unravel the toxic effects and mechanisms of Cd in immune cells and develop effective immunotherapies to mitigate its toxic effects.

## The role of n-3 PUFAs in Cd exposure

As mentioned previously, it is well known that Cd poisoning is extremely harmful to the body. Research on its treatment was initiated as early as the 20th century, and a wide variety of measures were adopted. As a result of in-depth research in recent years, the exploration of a wide range of antidotes to Cd poisoning has become a hot spot, including metal ion supplements, microorganisms, antioxidants, and so on (118–121). As new experimental advances continue to emerge, although more and more studies have proven that these methods can counteract Cd poisoning, problems such as inability to use them in practice or causing side effects of treatment inevitably arise. Therefore, n-3 PUFAs, a possible natural product as a potential treatment for Cd poisoning, is proposed here.

The earliest research on the interaction between n-3 PUFAs and Cd was conducted in 1997, when Howlett et al. explored the relationship between Cd and n-3 PUFAs in Saccharomyces cerevisiae (122). Their results showed that the cellular fatty acid unsaturation and plasma membrane would increase significantly when Cd enters Saccharomyces cerevisiae, especially DHA and EPA. In addition, compared with cells enriched in n-3 PUFAs, cells with low levels of n-3 PUFAs shows a more pronounced decrease in their cell viability. Interestingly, the study also found that potassium efflux is much higher in cells enriched in n-3 PUFAs than in cells with lower levels. Although the author failed to make a reasonable analysis of this phenomenon, in light of recent research, we believe that protective effects of Cd ions may be responsible, and they also have a protective effect on the cardiovascular system. Unfortunately, limited by species and technology, the study did not address the doubt, but for sure, the study demonstrated some kind of interaction between n-3 PUFAs and Cd. There were few studies exploring this relationship after that. It was not until 2007 when another related study mentioned this relationship again that researchers began to look at its implications (123). The study sheds light on the permeability of the gut through DHA supplementation at different concentrations and finds a significant increase in the accumulation of Cd in DHA-supplemented cells compared with the normal human colon carcinoma cell line. Further research found that Cd is absorbed and processed through the paracellular pathway, which reconfirms the previous conclusion that the role of n-3 PUFAs may be closely related to the Na-K-Ca ATP channel.

### In vivo

Among all body organs, the brain is most susceptible to oxidative stress. As previously mentioned, Cd induces oxidative stress by enhancing ROS production and damages mitochondria as a result of various factors. A more serious concern is that elevated ROS levels can cause antioxidant systems to malfunction and lead to neurodegenerative diseases, including Alzheimer’s and Parkinson’s (124–127). In the face of this damage, can n-3 PUFAs play a role? According to many studies, Cd may entry the brain and exerts its effects through the blood-brain barrier as an enzyme protein that protects this barrier (128, 129). A study showed that supplementation with n-3 PUFAs could increase monoamine oxidase and acetylcholinesterase levels in the body, and these indicators even return to near-normal levels in Cd-exposed mice, demonstrating that n-3 PUFAs have the potential to counteract Cd exposure (130). In addition, a study showed that, by supplementing ALA in Cd poisoned mice, the originally elevated ROS levels in the brain are alleviated, and n-3 PUFAs are found to further reduce neuroinflammation and neurodegeneration through regulated Nrf2/HO-1/JNK signaling pathway (131). It should be added that another study reported similar findings, albeit not for the damage caused by Cd, specifically demonstrating that ALA has an anti-apoptotic effect (132). Therefore, further studies are necessary to gain a deeper understanding of the mechanistic effects of ALA on Cd-induced oxidative stress and neurodegeneration.

As for the hippocampus, a part of the limbic system and located in the medial temporal lobe of the brain, has been shown to play an important role in learning and memory (133, 134). A study in rats showed that after Cd exposure, the number of neurons near the hippocampus would significantly reduce with the appearance of edema around the nerve, and training memory would significantly diminish, and after DHA supplementation, the memory function and hippocampal structure of the rats are improved (135). Interestingly, the study also found that the content of DHA decreases significantly as Cd enters the brain, but does not make quantitative tests to fully explain the whole process of this phenomenon. We suppose that this phenomenon may be explained by indirect involvement of n-3 PUFAs in pro-oxidative, pro-inflammatory, and memory-disrupting effects. In addition to this, several studies have been conducted on a range of antioxidant enzymes with positive results, showing significant decreases in the levels of SOD, CAT, and GST after exposure to Cd (136, 137). However, after supplementation with n-3 PUFAs, they all recover to varying degrees. Overall, based on the available studies, it is conceivable to consider supplementation of n-3 PUFAs as a possible medical adjunct in the suppression of inflammatory brain injury caused by Cd exposure.

In the liver, different studies have reported the effects of Cd exposure on oxidative stress and fatty acid composition, and the results consistently show changes across different animals, with the most important change in FAs composition being the reduction in the percentage of DHA in the body. Although the antioxidant system apparently responds to ROS generation under Cd exposure, it appears that the body’s own antioxidant capacity is insufficient to counteract cellular damage. Supplementation of n-3 PUFAs has a positive effect on the body’s antioxidant defense system, lipid peroxidation, and oxidative damage. It is pivotal to emphasize here that a study evaluating therapeutic concentrations showed that, n-3 supplementation could reduce liver tissue and cell damage from Cd exposure only for Cd exposure below 1 μM, whereas the antagonistic effect of supplementation with n-3 PUFAs is not obvious after Cd exposure above 1 μM (138). Therefore, although some studies have suggested that n-3 PUFAs can be used as a primary treatment for fatty liver disease, n-3 PUFAs may not be a primary treatment for liver disease caused by acute Cd exposure. However, we suggest exploiting preventive and rehabilitative therapeutic roles of n-3 PUFAs in the liver. Existing studies have shown that n-3 PUFAs supplementation can effectively resist oxidative damage caused by long-term chronic Cd exposure and have anti-inflammatory and anti-apoptotic effects (139–141). Studies have shown that pretreatment with n-3 PUFAs in stressed mice can significantly reduce subsequent stress-induced liver injury (142). Therefore, supplementing n-3 PUFAs in daily life is a good preventive measure. In addition, conventional drugs in the treatment of liver disease may have adverse effects, and their efficacy and safety are also questionable, while as natural products, n-3 PUFAs can be safely used. At the moment, we are predominantly concerned about the ratio and dosage, which do not appear to have a unified answer. In addition, it would be worthwhile to explore the possibility to gain a deeper understanding of the specific mechanisms of n-3 PUFAs in response to Cd exposure from genomic, proteomic, and metabolomic analysis.

In terms of the reproductive system, identification of factors that affect fertility has important clinical and public health implications. Many studies have pointed out that Cd exposure affects fertility (143–145), and FAs, as important substrates for early reproductive events, have received much attention. Human and animal studies have shown that ingestion of n-3 PUFAs through diet or dietary supplements can be effective in reducing the risk of early preterm birth, and n-3 PUFAs supplementation during pregnancy could also reduce the risk of allergic disease in childhood (146, 147). At the same time, supplementation of n-3 PUFAs in men also has a protective effect on their reproductive systems (148–150). In response to damage from Cd exposure, n-3 PUFAs are found to have positive effects on hormones that control reproduction (151–153). Serum testosterone and LH concentrations significantly go up after adding n-3 PUFAs to the diet of male model animals, thus we reckon the mechanisms behind these effects are related to the antioxidant properties of n-3 PUFAs. Therefore, it is reasonable to believe that n-3 PUFAs have ameliorating effects on Cd exposure-induced reproductive impairment. Indeed, significant reductions in sperm count, sperm motility, and the percentage of sperm with normal morphology were studied in Cd exposure-induced mice, and supplementation with n-3 PUFAs could largely alleviate the effects of Cd on semen quality (154). Researchers in another study thought that this improvement might be mediated by the antioxidant properties of n-3 PUFAs (155). As observed in all results, Cd exposure in both sexes decreased n-3 PUFAs levels in the gonads. Unfortunately, few studies have specifically investigated the effects of n-3 PUFAs on Cd exposure in the female reproductive system (156), which we think may be related to different metabolic demands during the reproductive period. However, a study pointed out that the concentration of n-3 PUFAs in the maternal placenta is significantly lower than the normal tissue level, which may explain why Cd can be transmitted through the placenta (157). In summary, although the reproductive system is not the primary target of Cd toxicity, n-3 PUFAs do counteract it to a certain extent, but the use of it as primary therapy requires further research, including specific action mechanisms in the male and female reproductive system.

The process of fat metabolism is greatly affected by Cd exposure which greatly increases the incidence of diabetes. Do n-3 PUFAs play a role here? This is a long-standing controversial issue. Some studies have indicated that supplementation with n-3 PUFAs increases fasting blood glucose, but changing the intake of n-3 PUFAs does not alter diabetes prevalence or have a therapeutic effect (158, 159). In addition, n-3 PUFAs do not appear to provide any protection to the renal function of diabetic patients. The use of n-3 PUFAs supplements is therefore unsupported by many studies. However, there is convincing evidence that n-3 PUFAs can lower triglyceride levels *in vivo (* 160). A long-term study suggests that n-3 PUFAs supplementation can reduce triglyceride concentrations in people at risk for diabetes (158), confirming their preventive role in Cd exposure-mediated diabetes.

In cancer, n-3 PUFAs have been confirmed to exert anticancer effects by regulating the expression levels of transcription factors such as NF-κB, p53, and cyclooxygenase-2 (COX2) (161). Among the Cd exposure, the role of n-3 PUFAs in colorectal cancer is of great concern, as Cd toxicity is strongly associated with colorectal cancer. Studies have shown that Cd exposure leads to abnormal COX-2 expression in HT-29 cells (162), which is consistent with the signaling pathway of n-3 PUFAs against carcinogenesis. In both human and animal models, n-3 PUFAs have also been shown to effectively reduce the development of colorectal cancer (163).

### In vitro

More research has focused on *in vitro*. The first is the composition of the plasma membrane. Different studies have found changes in plasma membrane permeability and the increasing sensitivity to Cd when cells are supplemented with n-3 PUFAs. A study noted that with the addition of Cd, cell membrane order would significantly dwindle, especially the contents of n-3 PUFAs as well as levels of antioxidant enzymes (164). We consider that this can be counteracted by n-3 PUFAs, since glutathione peroxidase activity can block the production of antioxidant enzymes during normal cellular metabolism, and glutathione has been found to be the main cellular target or sequestration site of Cd. Therefore, the decreased levels of antioxidant enzymes in cells after Cd exposure partly reflect the depletion of glutathione in cells. Combined with existing research, it is evident that n-3 PUFAs can participate in the regulation of glutathione levels in cells, thereby repairing oxidative damage. Cellular sensitivity is similar to plasma membrane permeability, with one study suggesting that fish daily supplemented with n-3 PUFAs are more sensitive to Cd exposure (165). After Cd exposure, the ROS in macrophages, granulocytes, and lymphocytes would change significantly, which apparently comes from the effects of n-3 PUFAs on the immune system. Studies show that a DHA-rich diet enhances the expression of the immunoglobulin M (IgM) gene, while an EPA-rich diet induces transcriptional down-regulation of genes involved in the Toll/NF-κB pathway, which in turn suppresses pro-inflammatory cytokines and induces detrimental damage to cells (166–168). By supplementing n-3 PUFAs to respond more efficiently to pathogen infections, the body benefits in the context of Cd contamination. In conclusion, we re-emphasize that the best response to long-term chronic Cd exposure might be daily supplementation with n-3 PUFAs.

## Discussion and outlook

**Figure 5:**
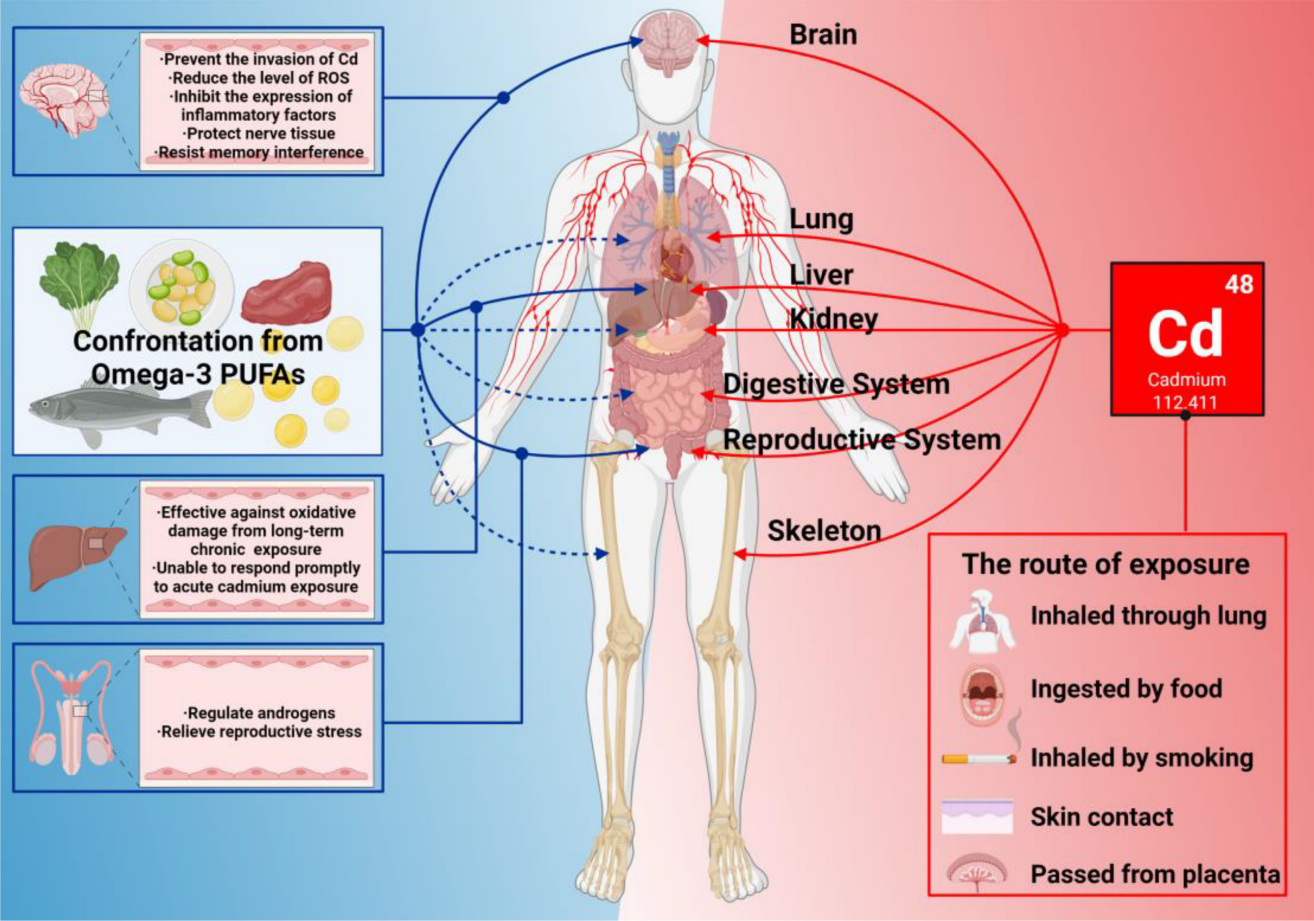
What n-3 PUFAs can do when Cd exposure harms bodies. In this figure, the red line represents the damage to various organs of the body caused by Cd exposure. The solid blue line represents the organ that has been studied so far with n-3 PUFAs that can resist damage from Cd exposure. The blue dashed line indicates that n-3 PUFAs are known to have a positive effect on the organ’s immune system.

It is always a priority for scientists to find ways to reduce the toxic effects of Cd, many of which are determined by its physical and chemical properties. Typically, Cd expresses pro-inflammatory activity, causing primary and secondary tissue damage by infiltrating innate immune cells (neutrophils, monocytes, and macrophages). The best defenses against Cd toxicity may be to inhibit ROS production, reduce oxidative stress levels, maintain redox balance, and inhibit abnormal immune signaling activation. Here we found that n-3 PUFAs are perfect for these tasks (**Figure 5**). It is worth noted that the relationship between n-3 PUFAs and Cd exposure is still complex from the current research. If bodyself has low levels of n-3 PUFAs, it would likely to fall into a vicious cycle of Cd exposure, producing less n-3 PUFAs but consuming more n-3 PUFAs. On the other hand, at present, most studies agree it is unreasonable to supplement n-3 PUFAs in related inflammatory diseases, and mainly because of the inability to adjust the internal ratio of n-3 PUFAs, the external ratio of n-3/n-6, and the possible lipid peroxidation. Recently, more research has begun to focus on this problem and provide a solution, along with the administration of other nutrients and drugs that may have oxidative or antioxidant effects. It is unfortunate that this solution is not yet perfect, and reducing the impact of these variables would possibly be one of the future research directions. However, n-3 PUFAs have been shown to play a significant role in rehabilitation therapy. Among the different research findings, we recommend the selection of supplemental doses up to 4.4 g/d.

Current research shows, n-3 PUFAs control the expression of a great variety of genes through different transcriptional factors, such as SREBP (169), PPARs (170), ChREBP (171), and NF-κB (172), which mainly regulate target gene transcription that encodes proteins involved in lipid and carbohydrate metabolism, thermogenesis, and inflammatory processes. However, it is important to point out that further studies are needed to elucidate these roles and to better understand the beneficial role of n-3 PUFAs in the mechanism of Cd exposure-induced disease, and to probe into their function as protective nutrients, aiming to prevent or treat the development of Cd exposure-related diseases.

## Author contributions

ZC and QL contributed equally to this work. ZC, QL, JW, XC, KW, YNW, and YHW: Writing and Editing. ZC and ZY: Conceptualization, Editing and funding acquisition. All authors contributed to the article and approved the submitted version.

## Funding

This research was supported by the Jiangsu Agricultural Science and Technology Independent Innovation Fund (CX(21)3119).

## Conflict of interest

The authors declare that the research was conducted in the absence of any commercial or financial relationships that could be construed as a potential conflict of interest.

## Publisher’s note

All claims expressed in this article are solely those of the authors and do not necessarily represent those of their affiliated organizations, or those of the publisher, the editors and the reviewers. Any product that may be evaluated in this article, or claim that may be made by its manufacturer, is not guaranteed or endorsed by the publisher.
